# A Canine Gait Analysis Protocol for Back Movement Assessment in German Shepherd Dogs

**DOI:** 10.3390/vetsci7010026

**Published:** 2020-02-19

**Authors:** Elif Surer, Andrea Cereatti, Maria Antonietta Evangelisti, Gabriele Paolini, Ugo Della Croce, Maria Lucia Manunta

**Affiliations:** 1 Department of Modeling and Simulation, Graduate School of Informatics, Middle East Technical University, 06800 Ankara, Turkey; 2 Department of Biomedical Sciences, University of Sassari, 07100 Sassari, Italy; acereatti@uniss.it (A.C.); gabrielepaolini@gmail.com (G.P.); dellacroce@uniss.it (U.D.C.); 3 Veterinary Department, University of Sassari, 07100 Sassari, Italy; maevangelisti@uniss.it (M.A.E.); lmanu@uniss.it (M.L.M.)

**Keywords:** gait analysis, german shepherd dogs, back movement, kinematics, kinetics, normative data

## Abstract

Objective—To design and test a motion analysis protocol for the gait analysis of adult German Shepherd (GS) dogs with a focus in the analyses of their back movements. Animals—Eight clinically healthy adult large-sized GS dogs (age, 4 ± 1.3 years; weight, 38.8 ± 4.2 kg). Procedures—A six-camera stereo-photogrammetric system and two force platforms were used for data acquisition. Experimental acquisition sessions consisted of static and gait trials. During gait trials, each dog walked along a 6 m long walkway at self-selected speed and a total of six gait cycles were recorded. Results—Grand mean and standard deviation of ground reaction forces of fore and hind limbs are reported. Spatial-temporal parameters averaged over gait cycles and subjects, their mean, standard deviation and coefficient of variance are analyzed. Joint kinematics for the hip, stifle and tarsal joints and their average range of motion (ROM) values, and their 95% Confidence Interval (CI) values of kinematics curves are reported. Conclusions and Clinical Relevance—This study provides normative data of healthy GS dogs to form a preliminary basis in the analysis of the spatial-temporal parameters, kinematics and kinetics during quadrupedal stance posture and gait. Also, a new back movement protocol enabling a multi-segment back model is provided. Results show that the proposed gait analysis protocol may become a useful and objective tool for the evaluation of canine treatment with special focus on the back movement.

## 1. Introduction

Instrumented movement analysis provides a powerful non-invasive tool to improve the diagnostic processes and to assess the efficacy of clinical interventions [1,2,3]. In the last decades, several two-dimensional (2D) and three-dimensional (3D) gait analysis protocols (GAPs) have been proposed for the analysis of the gait of dogs based on the use of video-based technology [2]. Gait patterns and force related characteristics were investigated in dogs for several clinical applications given that kinetics and kinematics studies help to clarify the effects of therapeutic choices and to objectively quantify the outcome after treatment.

In 2D GAPs, the sagittal joint kinematics are provided under the hypothesis that the dog’s sagittal plane coincides with the plane identified by the vertical axis of the global frame and the direction of progression (DoP) of the dog [4,5]. The main advantage of 2D GAPs lies in the use of a single video camera and lower number of skin markers attached to the dog [6].

Conversely, 3D GAPs require the use of multiple cameras arranged to define a proper measurement volume. The latter can be further classified depending on whether the body segments under analysis are modeled as rigid bodies or line segments [7,8]. The rigid body representation allows for a six degrees of freedom joint kinematics description (three angular and three linear displacements) [9]. However, the joint translation estimates are normally discarded in human movement analysis [10] since they are not sufficiently reliable—mainly due to the presence of soft tissue artefacts [11]. For a complete description of the body segment orientation in the 3D space, at least three non-aligned markers for each segment are needed. The latter requirement entails the use of a more complex marker-set, which in turn implies a longer preparation of the subject and a decrease in the natural movement of the subject. When each body segment is modeled as a line segment, only two markers for each segment are required [12,13,14,15,16]. A limitation associated to this simplified approach is that rotations around the segment axis are neglected. The joint angular kinematics can be computed as the angle between 3D vectors [6,8] or, more intuitively, by determining the angles resulting from their projections on the sagittal, frontal and horizontal planes [16,17].

The assessment of gait deviations in pathological populations is commonly performed by comparing a patient’s motion data with normative reference data collected from a matched sound group. The comparison is carried out through few meaningful indices either computed over an interval of time or extracted from it. Assessing their repeatability and confidence interval is crucial to identify the limits of physiological gait variability and those of pathological gait. This is even more critical in canine gait analysis given the significant role of soft tissue artifacts and high morphometric and biomechanical variability characterizing different breeds [18,19]. Therefore, the availability of breed-specific normative data is key to assess the severity of gait pathologies.

Force platform data have also been used to evaluate orthopedic and neurological diseases in dogs. Souza et al. [20] measured and evaluated the ground reaction forces of peak vertical force and vertical impulse in the pads of GS dogs using a pressure sensitive walkway for both fore and hind limbs. Static acquisitions’ kinetics data give insight on the healthy dogs’ quadruple posture and these data can further be useful to provide a basis for comparison with pathological dogs. For example, Souza et al. [21] assessed the vertical forces according to radiographic hip grade in GS dogs and made several comparisons taking into consideration the lameness degrees of hip dysplasia and considering the data of healthy dogs as reference.

The majority of the GAPs proposed in the literature was devised for the analysis of the hind and forelimbs kinematics [2]. Only a few of studies focused on the back movements in spite of its importance in the veterinary sciences [6,12,16].

The aim of this study is twofold: (i) to present and validate a canine GAP specifically designed for the evaluation of the back movement in the sagittal and horizontal planes; and (ii) to provide preliminary normative data (bootstrapping confidence interval measures) of the spatial-temporal parameters, joint kinematics and kinetics quantities during quadrupedal stance posture and level gait in healthy GS dogs.

## 2. Materials and Methods

### 2.1. Animals

Eight healthy large-sized adult GS dogs, four males and four females, were enrolled in the study. Mean (± SD) body weight and age were 38.8 ± 4.2 kg and 4 ± 1.3 years, respectively. Animals were considered eligible for the study if they were two years of age or older, without pathologies identified on the basis of complete physical examinations and radiographic evaluations of the coxofemoral and femorotibial joints. All dogs were owned animals; a written informed consent was obtained by their owners. Since the testing protocol involved no invasive procedures or discomfort to the dogs, ethical approval was granted by the University of Sassari internal ethical review board (protocol no. 1108 3/24/2015).

### 2.2. Marker-Set and a Multi-Segmental Skeletal Model

**Figure 1:**
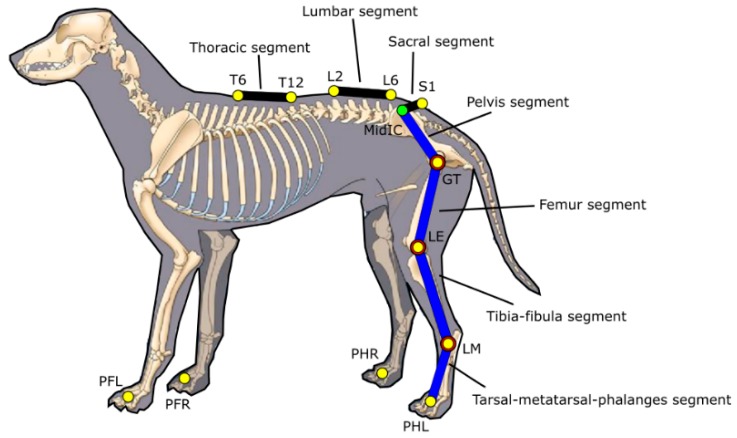
Marker-set of 17 retro-reflective spherical markers attached to selected ALs; the multi-segmental model included 10-line segments including three segments for modeling the back (sacral segment, lumbar and thoracic segments). Tarsus, stifle and hip joints are modeled as spherical hinges.

The marker-set consisted of 17 retro-reflective spherical markers (∅ 14 mm) placed on the following anatomical landmarks (ALs): dorsum of each paw (Left forelimb paw: PFL, right forelimb paw: PFR, left hind limb paw: PHL, right hind limb paw: PHR), on the right and left lateral malleolus (RLM, LLM), on the lateral aspect of the right and left femoral distal epiphysis (RFE, LFE), right and left greater trochanters (RGT, LGT), right and left iliac crests (RIC, LIC), 1st spinal process of sacrum (S1), 2nd and 6th spinal processes of lumbar vertebrae (L2, L6), 6th and 12th spinal processes of thoracic vertebrae (T6, T12). The position of a virtual marker (MidIC)—defined as the midpoint between the two ICs—was also computed (Figure 1).

Marker locations on the spine were chosen so that data on the physiological spine movements characterized by major flexion/extension, lateral bending and axial rotation in the thoraco-lumbar transition area [12] and in L7-S1 segments, could be collected [22].

**Figure 2:**
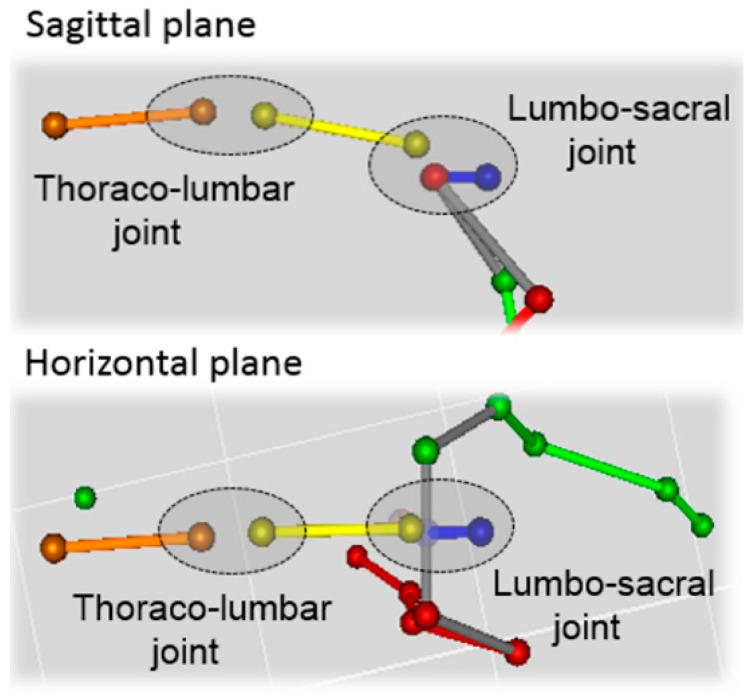
The locations of the thoraco-lumbar and lumbo-sacral joints in sagittal and horizontal planes.

A multi-segment model including 10-line segments was defined from the marker positions: right and left tarsal-meta-tarsal-phalanges (Pes) segments (PH-LM), right and left tibia-fibula segments (LM-LE), right and left femur segments (LE-GT), a pelvis segment (GT-MidIC), a sacral segment (S1-MidIC), a lumbar segment (L6-L2) and a thoracic segment (T12-T6). In addition, the model incorporates the assumption that tarsus, stifle and hip joint are spherical (Figure 2).

### 2.3. Kinematics Variables

Gait cycles were identified with the position of forelimbs only, with respect to the ground by manually labeling the contact points (initial (IC) and final contacts (FC)) using the Vicon Nexus’ keyframing tool. The stride duration was defined as the time interval between two successive ICs of the same paw. As in [23], one step was defined and calculated from FC to IC. The keyframes of FC and IC events were identified visually and were marked using Vicon’s Nexus software and the step time was calculated automatically by the software based on those marked key events. Stride and step lengths were defined as the distances along the DoP traversed by the markers attached on the dorsum of the forelimb paw between the corresponding intervals of time.

The joint angular kinematics were determined by projecting the markers’ position vectors onto the sagittal and horizontal planes and then by computing the relevant planar angles. The sagittal plane was defined by the DoP and the vertical axis while the horizontal plane was orthogonal to the vertical axis of the global frame. Sagittal angular kinematics were estimated for the tarsal joints’ flexion-extension (flex/ext), the stifle joint (flex/ext), the hip joint (flex/ext); the pelvis motion was described in the sagittal plane (pelvic tilt) and the horizontal plane (pelvic rotation). The movement of the vertebral column was described by the relative angular motion in the sagittal and horizontal planes between the sacral and lumbar segments (lumbar-sacral joint) and the lumbar and thoracic segments (thoraco-lumbar joint).

### 2.4. Experimental Procedure

Skin markers were attached to the dogs’ skin using epoxy resin glue. The hair was clipped prior to the marker placement to reduce the motion of the markers with respect to the skin.

Instantaneous positions of the markers were reconstructed using a six-camera Vicon T-20 (Oxford Metrics, UK) stereo-photogrammetric system acquiring at 100 frames per second with a sensor resolution of 1600 × 1280 pixels. The measurement volume was 6 m× 6 m× 2 m. Two AMTI (Watertown, MA, USA) force platforms recorded ground reaction forces at 100 Hz sampling frequency. Measurement accuracy of the platforms were ±0.5% of applied load. Force platforms recorded ground reaction forces synchronously with the Vicon T-20 stereo-photogrammetric system. Markers’ coordinates were low-pass filtered using Butterworth filter (6 Hz cut-off frequency) to eliminate the noise while calculating the trajectories.

The experimental acquisition session consisted of static trials with the dog maintaining a quadrupedal stance posture during which both stereo-photogrammetric and force data were collected and gait trials during which only stereo-photogrammetric data were acquired.

While the dog was maintaining a reproducible stance posture, three separate static conditions were collected to provide a complete posturographic assessment based on recordings provided by the two force platforms: (a) left and right forelimb paws on platforms 1 and 2, respectively; (b) left and right hind limb paws on platforms 1 and 2, respectively, (c) left and right hind paws on platform 1 and left and right for paws on platform 2.

Before gait recordings, the dogs were familiarized with the walkway by being walked back and forth several times. This procedure allowed the handler to determine each dog’s natural gait speed. During the acquisition, each dog was held on a leash and walked along a 6 m long walkway at the natural speed. A total of six gait cycles (three right and three left) were recorded during the same acquisition session.

### 2.5. Data Analysis

#### 2.5.1. Static Trials

**Figure 3:**
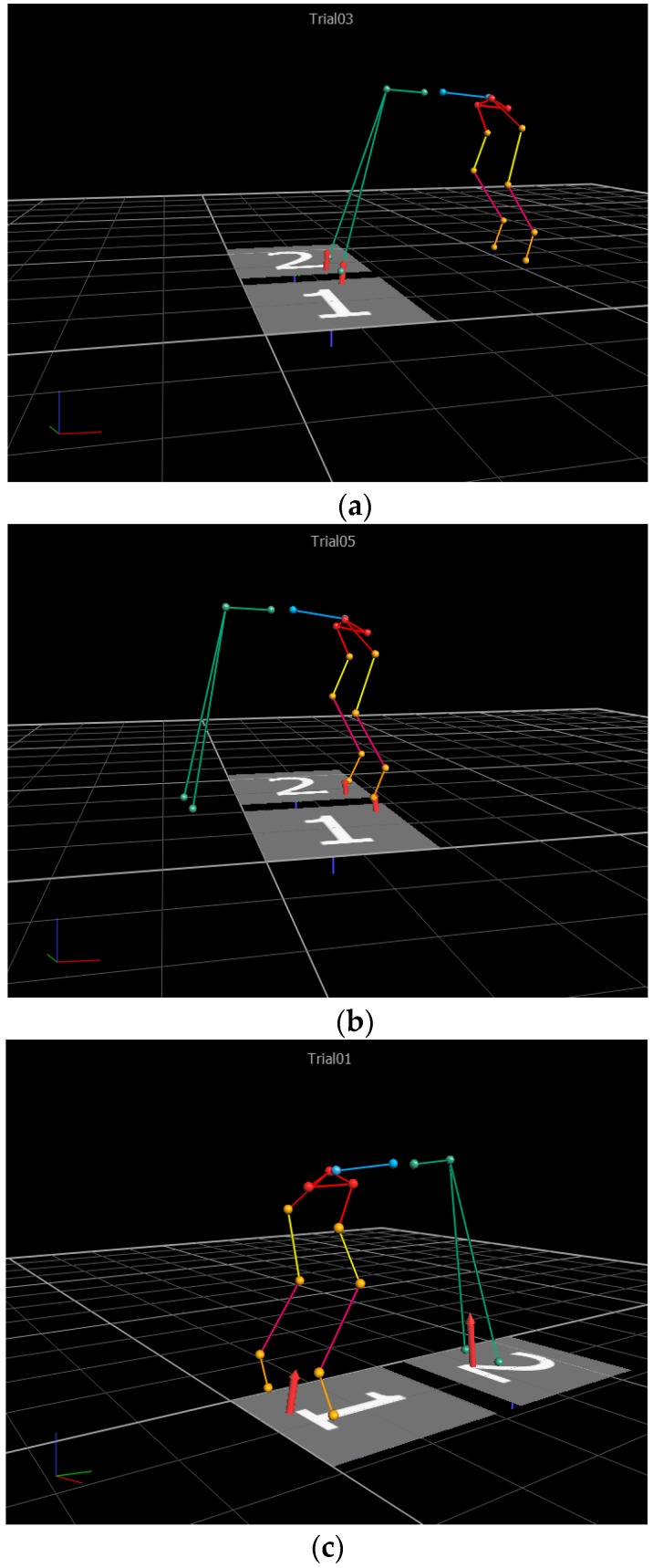
Force platform acquisitions and screenshots on static acquisitions from the Vicon Nexus’ software: (**a**) left and right forelimb paws on platforms 1 and 2, respectively; (**b**) left and right hind limb paws on platforms 1 and 2, respectively, (**c**) left and right hind paws on platform 1 and left and right for paws on platform 2.

For the three static acquisitions (Figure 3), for each dog, the mean values of the vertical ground reaction forces normalized to the dog’s weight (VGRF), were computed over a duration of 10 s. Grand mean (Gmean) values of the normalized ground reaction forces along with the corresponding standard deviation values of the means (stdm) were computed over the static acquisitions from the dogs (See Supplementary Material Table S1 to access the normative data of force platforms, for all subjects). Gmean and stdm values of the postural joint and segmental angles were also computed for the three cases mentioned above given that they have the potential to be used as a reference for gait angles.

#### 2.5.2. Gait Trials

For each dog, mean and std values of the spatial-temporal parameters over six gait cycles were computed. For each parameter, the Gmean, the corresponding stdm value and the coefficient of variation (CV%) were then computed over the samples of subjects.

Joint kinematics timings during gait were expressed as percentage of the gait cycle and, for each dog, mean and std values were computed for each gait cycle over six gait cycles. Then, for each kinematics variable, Gmean and stdm values were computed over the samples of subjects for each percentage value of the gait cycle. From the grand mean angular curves, the range of motion (ROM) was computed (i.e., the difference between the maximum and minimum values). To determine the range of normal gait and to better measure deviations from the normative band, confidence interval bands and mean population estimates across gait cycles were calculated for sagittal and horizontal measurements of hip, tarsal and stifle joints from Bootstrap Upper Confidence Interval (UCI) and Bootstrap Lower Confidence Interval (LCI) values [24,25,26].

## 3. Results

Amplitude of the ground reaction forces during static acquisitions for the forelimb and hind limb paws are reported in Table 1.

The values of the spatial-temporal parameters averaged over gait cycles and subjects are reported in Table 2.

**Figure 4:**
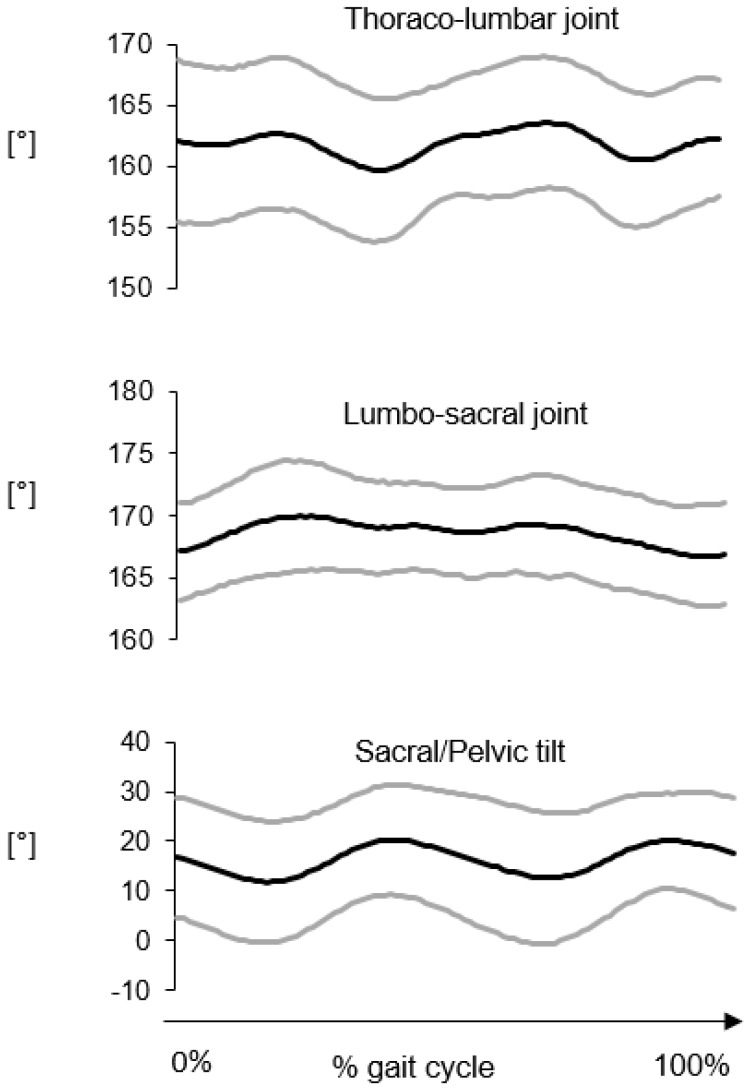
Thoraco-lumbar and lumbar-sacral joint kinematics in the sagittal plane. The average curve (black line) ± the standard deviation (grey lines) were computed over six gait cycles for each subject then averaged for 8 subjects.

**Figure 5:**
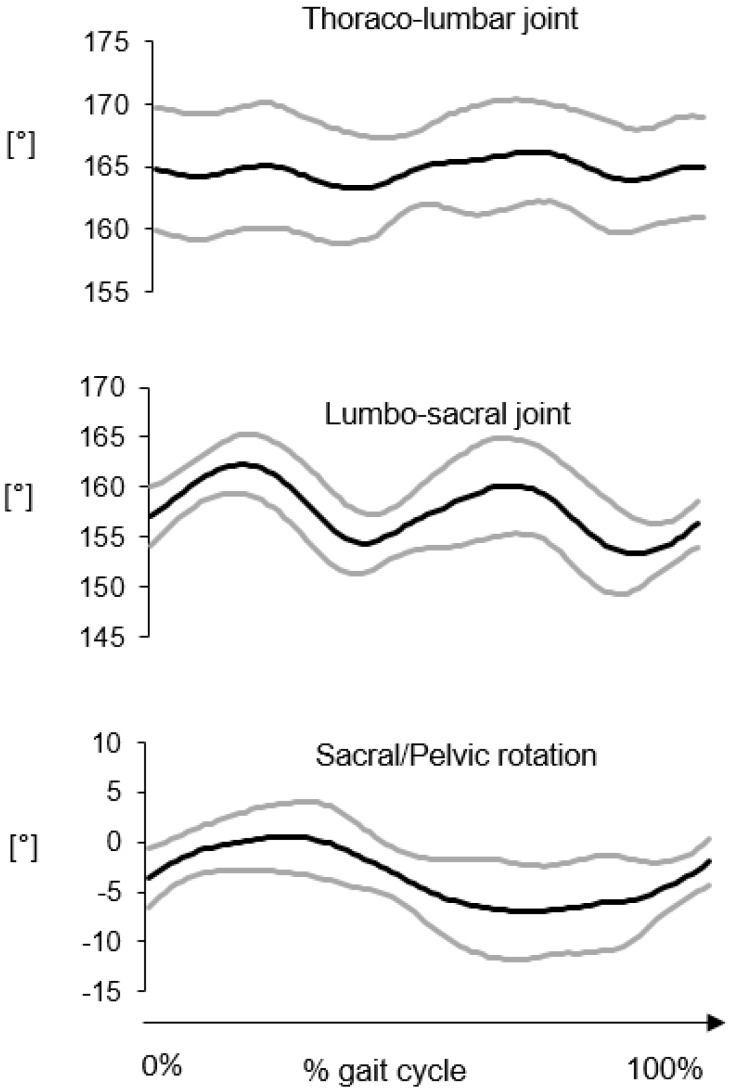
Thoraco-lumbar and lumbar-sacral joint kinematics in the horizontal plane. The average curve (black line) ± the standard deviation (grey lines) were computed over six gait cycles for each subject then averaged for 8 subjects.

**Figure 6:**
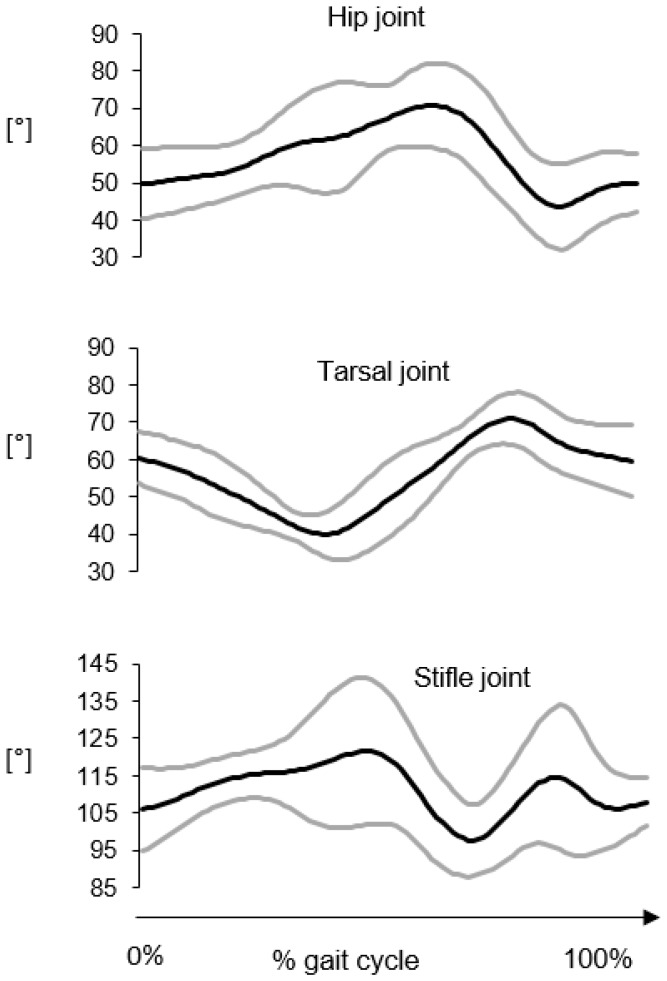
Hip, stifle and tarsal joint kinematics in the sagittal plane. The average curve (black line) ± the standard deviation (grey lines) were computed over six gait cycles for each subject then averaged for 8 subjects.

Average kinematic curves in the sagittal and horizontal planes for the thoraco-lumbar and lumbar-sacral joints are reported in Figure 4 and Figure 5, respectively. The average flexion-extension joint kinematics for the hip, stifle and tarsal joints are reported in Figure 6. The average ROM values are reported in Table 3.

As expected in gait, the largest angular excursions were observed in the sagittal plane, in descending order from the hip joint (ROM ≈ 31°) to the tarsal joint (ROM ≈ 24°). The angular motion between the sacral and pelvis segments presented similar values both in the sagittal and horizontal planes (≈7°–9°). The smallest joint movements were observed for the thoraco-lumbar joint both in the sagittal and horizontal planes and the lumbar-sacral joint in the sagittal plane (≈3°–4°). Interestingly, a larger range of motion occurred at the lumbar-sacral joint in the horizontal plane (≈9°).

Mean values and 95% percentile CI values for the estimated kinematic quantities are provided to be used as normative data (see Supplementary Material Table S2 to access the mean values and CI calculations and Table S3 to access the grand mean and standard deviation of all subjects’ joint kinematics values).

## 4. Discussion

In this study, a 3D GAP to evaluate the movement of the forelimbs, hind limbs and spine in healthy GS dogs, is presented. To the best of authors’ knowledge, this is the first study reporting normative values of the spatial-temporal parameters, kinematics and kinetics quantities during quadrupedal stance posture and level gait in healthy GS dogs in terms of bootstrapping confidence interval measures. The availability of normative data acquired from healthy dogs is critical to properly compare the outcomes of orthopedic and neuro-orthopedic treatments. The gait of dogs may be expected to vary among breeds with different conformation. It is recognized that some breeds of dog are predisposed to certain neuro-musculoskeletal pathologic conditions. However, given the differences in conformation among breeds, it may be expected that inherent biomechanical differences in joint function are responsible for alterations in joint loading patterns that may contribute to susceptibility to specific injuries or degenerative processes [27]. The availability of normative data acquired from healthy dogs is critical to properly compare the outcomes of orthopedic and neuro-orthopedic treatments. Although numerous scientific studies have been conducted on gait analysis in dogs, few of them analyses the biomechanical aspect of the thoraco-lumbar spine. Many questions remain open regarding the biomechanical function of the lumbar portion of the vertebral column where important debilitating pathologies develop, particularly in the GS dogs.

As expected in gait, 3D GAP showed the largest angular excursions in the sagittal plane, in descending order from the hip joint to the tarsal joint. Regarding the vertebral column movements, the thoraco-lumbar joint had the smallest joint movements both in the sagittal and horizontal planes. ROM in sagittal plane increases from thoraco-lumbar to lumbar-sacral joint.

Thoraco-lumbar lower ROM in sagittal plane may be explained by the stabilizing effect of the back muscles, while the horizontal joint movement could be due to the contraction of the longissimus dorsi muscle.

This study’s in vivo findings agree with an in vitro experiment describing the three-dimensional motion pattern of the caudal lumbar and lumbosacral portions of frozen specimen from vertebral column of GS dogs, according to which motion in sagittal planes increased from L5-6 to L7-S1. The increased ROM in sagittal plane at the lumbar-sacral joint could be possible through the vertically placed articular facets between L7 and S1 and the firm attachment of the hind limb musculature to the pelvis, which also has firm articulation to the fused sacrum, and so a forward–upward motion is provided [12]. The high mobility of the lumbosacral joint at a level where all the loading forces from the pelvis, sacroiliac joint, and sacrum are transmitted to the lumbar portion of the vertebral column may predispose to high wear and tear and may be a risk factor for disk degeneration. However, lumbar portions of the vertebral column of GSDs are reported as less flexible at L7-S1 than those of dogs of other breeds. Therefore, it is difficult to explain why GSDs are predisposed to Degenerative Lumbosacral Stenosis (DLSS) since in this breed, mobility in the lumbosacral junction is smaller, compared with dogs of other breeds.

For each gait cycle, confidence interval bands and mean population estimates were calculated for sagittal and horizontal measurements of hip, tarsal and stifle joints (see Supplementary Material Table S2 to access the mean values and CI calculations). Bootstrap estimate of lower and upper range of confidence interval, and sample estimate of population mean are provided for each subject and for all subjects to be used as baselines for future studies. It is fundamental to highlight that, due to the presence of soft tissue artefacts generated by the sliding of the skin markers over the bony prominences and of limited joint angular excursions observed for the back complex, the signal-to-noise ratio of the angular kinematic quantities describing the back motion is expected to be low. Furthermore, it is important noting that for reducing the number of markers and thus simplifying the experimental protocol, only two markers per segment were used. This choice implied that joint kinematics is computed from the projection of the line segments (3D vectors) onto the anatomical planes by disregarding the full 3D joint kinematics (e.g., Euler angles) (See Supplementary Material Table S4 to access the normative data of joint kinematics, for all subjects). Despite the limitations mentioned above, the adoption of a multi-segment back model for the gait analysis and adding a virtual marker (MidIC) enables the protocol to provide a quantitative description of absolute angles of the vertebral column during both quadrupedal stance posture and gait —which can be clinically valuable since no normative data are available in the literature.

Outcomes of the recent studies on measuring and evaluating the ground reaction forces [20,21,22,23,24,25,26] demonstrate the importance of providing normative data and we are providing the most crucial parameters related to kinetics —anterior, posterior and anterior posterior vertical ground reaction forces normalized by body weight (See Supplementary Material Table S1 to access the normative data of force platforms, for all subjects). Static acquisitions measure the quadrupedal posture and the results of this study from healthy dogs can be used as a baseline to measure the amount of deviation from the normative data for pathological dogs.

Spatio-temporal parameters’ values were compared for mean, standard deviation and ROM values (see Supplementary Material Table S5 to access the normative data of spatio-temporal parameters, for all subjects). Further kinematic studies including dogs with back pain are necessary to establish the significance of these observations.

In the literature, stance duration parameter in terms gait cycle (% cycle) was presented for normal and post-hemilaminectomy (PHL) Dachshunds [28] for affected and contralateral sides (Control group: Affected 22.4 ± 1.4 Contralateral 23.0 ± 1.4; PHL dogs: Affected 26.8 ± 1.7 Contralateral 26.2 ± 1.7). Besides, Light et al. [29] presented temporal-spatial gait analysis of healthy Labrador Retrievers for stance phase on a portable walkway system—stride length, stride time, stance time and stance time percentage. The only common measurement of this study with Light et al. was stride length and the results were similar: Mean stride length of Light et al.’s study was 0.88 cm while in this study stride length ranged between 80 cm to 110 cm with a standard deviation of 12 cm. Similar to Sutton et al. [28], Miqueleto et al. [30] presented stance and swing-related parameters of healthy and hip-dysplastic GS dogs. In Miqueleto et al.’s study, the speed of the dogs was determined by the treadmill speed and the dogs were trotted between 2.1 and 2.2 m/s while in this study, the healthy GS dogs preserved a natural speed having 1.3 m/s speed. Thus, to the authors’ knowledge, this is the first study reporting normative values of a complete set of spatial-temporal parameters (i.e., cadence, stride time, step time, stride length, step length and walking speed) for healthy GS dogs.

Normative biomechanics parameters in GS dogs are necessary since they provide preliminary information for the evaluation of affected animals given that kinetic and kinematic data can vary among and within dog breeds. The proposed back movement GAP was specifically designed to describe the movement of the vertebral column which was divided into three main rigid segments as opposed to previous studies in which the spinal column was modeled as a single segment. It is expected that the same protocol could be used to assess functional limitations and motor impairments associated to the presence of pathologies affecting the vertebral column and in particular, Degenerative Lumbosacral Stenosis (DLSS) disease in dogs [31].

## 5. Conclusions

The study proposes a new back movement protocol which is applicable to other breeds and to assess gait deviations and it provides the relevant normative data for kinematics, kinetics and spatial-temporal variables complemented with a statistical description.

Future studies are needed to verify the efficacy of the method described in this study on affected animals before its routine clinical use.

## Figures and Tables

**Table 1:** Vertical ground reaction forces normalized to the dog’s weight computed for three different conditions, for a 10 s duration. Grand mean values and standard deviation of the means values over subjects are reported.

**Conditions**	**Anterior Only**			
**Sub Conditions**	**Right Paw**		**Left Paw**	
**VGRF**	**Gmean**	**Stdm**	**Gmean**	**Stdm**
	−23.1	0.7	−34.9	0.6
**Conditions**	**Posterior Only**			
**Sub Conditions**	**Right Paw**		**Left Paw**	
**VGRF**	**Gmean**	**Stdm**	**Gmean**	**Stdm**
	−16.5	0.2	−19.7	0.2
**Conditions**	**Anterior and Posterior**			
**Sub Conditions**	**Hind Paws**		**Fore Paws**	
**VGRF**	**Gmean**	**Stdm**	**Gmean**	**Stdm**
	−38.7	0.4	−57.2	0.7

**Table 2:** Spatial-temporal parameters: grand mean, standard deviation of the means coefficient of variation (CV%) over subjects and gait cycles.

Spatial-Temporal Parameters	Gmean	Stdm	CV%
**Cadence [step/min]**	158.7	21.72	14
**Stride time [s]**	0.8	0.08	11
**Step time [s]**	0.4	0.07	19
**Stride length [m]**	1.0	0.12	12
**Step length [m]**	0.5	0.05	10
**Walking speed [m/s]**	1.3	0.33	25

**Table 3:** Minimum (min), maximum (max), range of motion (ROM) joint kinematics values (in degrees (°)) averaged over gait cycles and subjects and their standard deviation values for 9 different joints with respect to the planes reported in parentheses.

Joint Kinematics		Min [°]	Max [°]	ROM [°]
**1**	**Thoraco-lumbar (sagittal)**	160	164	4
**2**	**Thoraco-lumbar (horizontal)**	163	166	3
**3**	**Lumbar-sacral (sagittal)**	167	170	3
**4**	**Lumbar-sacral (horizontal)**	153	162	9
**5**	**Sacral/Pelvic tilt (sagittal)**	12	20	8
**6**	**Sacral/Pelvic rotation (horizontal)**	−7	0	7
**7**	**Hip joint (sagittal)**	40	71	31
**8**	**Stifle joint (sagittal)**	44	71	27
**9**	**Tarsal joint (sagittal)**	98	122	24

## Supplementary Materials

The following are available online at http://www.mdpi.com/2306-7381/7/1/26/s1, Table S1: Normative Force Platform Data of All Subjects (Excel file). Table S2: Mean and Confidence Interval Calculations (Excel file). Table S3: Grand mean and Stdev Calculations of Joint Kinematics (Excel file). Table S4: Normative Data of All Subjects’ Joint Kinematics (Excel file). Table S5: Normative Spatio-Temporal Data of All Subjects (Excel file).
